# The prognostic value of gastrointestinal bleeding in gastrointestinal stromal tumor: A propensity score matching analysis

**DOI:** 10.1002/cam4.2328

**Published:** 2019-06-13

**Authors:** Wenze Wan, Zhen Xiong, Xiangyu Zeng, Wenchang Yang, Chengguo Li, Yu Tang, Yao Lin, Jinbo Gao, Peng Zhang, Kaixiong Tao

**Affiliations:** ^1^ Department of Gastrointestinal Surgery, Union Hospital, Tongji Medical College Huazhong University of Science and Technology Wuhan China

**Keywords:** gastrointestinal bleeding, gastrointestinal stromal tumors, propensity score matching, tumor rupture

## Abstract

**Background and objectives:**

Whether gastrointestinal (GI) bleeding indicates gastrointestinal stromal tumor (GIST) rupture and impacts prognosis is unclear. We examined the prognostic value of GI bleeding in GIST.

**Methods:**

Primary GIST patients with (GB group) or without (NGB group) initial symptoms of GI bleeding were retrospectively studied. Propensity score matching (PSM) was conducted to reduce confounders.

**Results:**

Eight hundred patients were enrolled. Male gender [odds ratio (OR) = 1.517, *P* = 0.011], tumors in the small intestine (OR = 2.539, *P* < 0.001), and tumor size 5‐10 cm (OR = 2.298, *P* = 0.004) increased the odds of GI bleeding; age >60 years decreased the odds (OR = 0.683, *P* = 0.031). After PSM, 444 patients were included (222 in each group). Relapse‐free survival (RFS) (*P* = 0.001) and overall survival (OS) (*P* = 0.002) were both superior in the GB group. In subgroup analysis, the GB group achieved a superior RFS (*P* = 0.005) and OS (*P* = 0.007) in patients with small intestine GIST, but not stomach or colorectal GIST.

**Conclusions:**

GIST patients with age <60, male gender, tumors located in the small intestine, and tumors 5‐10 cm in size had a higher risk of GI bleeding. GIST patients with GI bleeding had a superior RFS and OS. This difference was statistically significant only in small intestine GIST.

## 1 INTRODUCTION

Gastrointestinal stromal tumor (GIST), with an annual incidence of 10‐15 cases per million people, is the most common mesenchymal‐derived tumor of the gastrointestinal (GI) tract.^1^, ^2^ About 69% of patients with GIST are symptomatic, and GI bleeding is the most common clinical symptom, presenting as hematemesis, hematochezia, or melena.^3^

GIST has a varying malignant potential ranging from small lesions with benign behavior to aggressive sarcomas.^1^ The modified National Institutes of Health (NIH) risk classification scheme, which encompasses 4 factors (size, mitotic count, site, and rupture), is frequently used to estimate the risk of recurrence after surgery.^4^ Notably, patients with tumor rupture, regardless of tumor location, tumor size, and mitotic count, are classified as high‐risk in the modified NIH criteria, because tumor rupture into the enterocoelia that occurs either spontaneously or during surgery increases the risk of tumor cell dissemination. Tumor rupture can predict survival independent of the size, mitotic count, and location of the tumor.^5^ However, whether GI bleeding indicates tumor rupture in the alimentary canal and can impact survival is unknown.

Our previous study demonstrated that, compared to GIST patients with GI bleeding, patients without GI bleeding showed an inferior relapse‐free survival (RFS).^5^ However, several recent studies have reported that GI bleeding is a negative prognostic factor.^6^, ^7^ However, these were both retrospective studies, and the reliability of these statistical results might be weakened because some characteristics that influenced prognosis were significantly different between patients who did and did not have GI bleeding.^6^, ^7^, ^8^ Hence, the prognostic impact of GI bleeding on GIST remains to be clarified. With the aim of achieving a more credible conclusion, the latest data from GIST patients in the Union Hospital, Tongji Medical College, Huazhong University of Science and Technology were collected, and propensity score matching (PSM), which can balance the covariates and confounders in nonrandomized studies,^9^ was performed. Moreover, subgroup analysis based on tumor location was conducted to further explore GI bleeding in GIST patients.

## 2 MATERIALS AND METHODS

### 2.1 Patients

Between January 2005 and December 2017, 1027 patients were diagnosed with primary GIST at the Union Hospital, Tongji Medical College, Huazhong University of Science and Technology. Among them, 800 patients were enrolled in this study. The exclusion criteria were as follows: (1) extra‐GI stromal tumors, (2) distant metastasis or invasion of the adjacent organs, (3) R1 or R2 resection, and (4) missing data and incomplete variables. Patients were divided into 2 groups: those who presented with GI bleeding (GB group) and those who presented without GI bleeding (NGB group). Demographic and clinicopathological data were collected and recurrent risk assessment was conducted according to the modified NIH criteria.^4^

**Figure 1:**
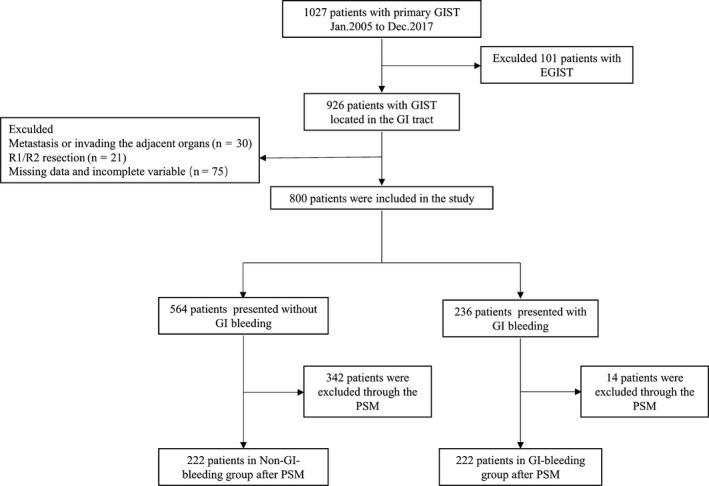
Patient selection flowchart

The flow chart for extracting eligible cases and grouping is demonstrated in Figure 1. This study was approved by the Ethics Committee of Tongji Medical College, Huazhong University of Science and Technology.

### 2.2 Follow‐up

Postoperative follow‐up was performed routinely (3‐6 months) by specially trained researchers. The follow‐up information, including adjuvant therapy, recurrence, and death were collected. The latest follow‐up date for the study was 1 July 2018. RFS was defined as the time from surgery to the first diagnosis of recurrent disease, and overall survival (OS) was defined as the time from surgery to death.

### 2.3 Propensity score matching

Propensity score matching was performed to eliminate the different distributions of covariates among individuals in the 2 groups. Seven covariates (age, gender, tumor location, tumor size, mitotic rate, tumor rupture, and adjuvant imatinib treatment) were selected to calculate the propensity score. Tumor rupture was strictly defined as tumor spillage or fracture, piecemeal resection, incisional biopsy, gastrointestinal perforation to the abdominal cavity, or blood‐tinged ascites. The PSM was conducted based on the logic of the propensity score and one‐to‐one nearest neighbor matching. The caliper was 0.02. The balance of covariates after matching was assessed using the standardized difference.^10^

### 2.4 Statistical analysis

Quantitative data were expressed as mean ± SD, and the differences were compared using an independent *t* test. The Chi‐square test or Fisher's exact test was used to compare differences in categorical data from different groups. Univariate and multivariate logistic regression models were constructed to explore the association of demographic and clinicopathological characteristics with GI bleeding. The survival curves were plotted by the Kaplan‐Meier method and the difference was compared by log‐rank test. SPSS software (SPSS 20.0, Chicago, IL, USA) was used for data management and statistical analyses. PSM was performed using Stata 14.0 (StataCorp, College Station, TX, USA). A 2‐tailed *P* < 0.05 was considered statistically significant.

## 3 RESULTS

### 3.1 Demographic data and clinicopathological characteristics

The characteristics of the entire cohort and the propensity score‐matched groups are shown in Table 1. Among the entire cohort, the median age was 56 years, and 436/800 (54.5%) of patients were men. According to the modified NIH classifications, 166/800 (20.8%) cases were classified as very low risk, 260/800 (32.5%) as low risk, 105/800 (13.1%) as intermediate risk, and 269/800 (33.6%) as high risk. Before PSM, significant imbalances in gender (*P* = 0.004), age (*P* = 0.008), tumor location (*P* < 0.001), tumor size (*P* = 0.009), recurrence risk (*P* = 0.011), and adjuvant imatinib therapy (*P* = 0.0037) were found between the 2 groups. After PSM, 222 patients comprised each of the 2 groups, and all baseline variables were more balanced (*P* > 0.05).

**Table 1:** Comparison of demographic and clinicopathological characteristics before and after propensity score matching

Characteristics	Overall population 【N = 800 (%)】	Before matching				After matching			
		GB^a^ group【N = 236 (%)】	NGB^b^ group【N = 564 (%)】	*χ* ^2^/*t*	*P*	GB group【N = 222 (%)】	NGB group【N = 222 (%)】	*χ* ^2^/*t*	*P*
Gender				8.188	0.004			0.037	0.847
Female	364 (45.5)	89 (37.7)	275 (48.8)			89 (40.1)	91 (41.0)		
Male	436 (54.5)	147 (62.3)	289 (51.2)			133 (59.9)	131 (59.0)		
Age(year)				7.064	0.008			0.100	0.752
≤60	528 (66.0)	172 (72.9)	356 (63.1)			158 (71.2)	161 (72.5)		
>60	272 (34.0)	64 (27.1)	208 (36.9)			64 (28.8)	61 (27.5)		
Tumor location				40.555	<0.001			1.442	0.486
Stomach	474 (59.3)	105 (44.5)	369 (65.4)			105 (47.3)	107 (48.2)		
Small intestine	284 (35.5)	123 (52.1)	161 (28.6)			109 (49.1)	102 (45.9)		
Colorectum	42 (5.2)	8 (3.4)	34 (6.0)			8 (3.6)	13 (5.9)		
Tumor size(cm)				9.431	0.009			0.047	0.977
≤5	479 (59.9)	133 (56.4)	346 (61.3)			119 (53.6)	121 (54.5)		
5‐10	223 (27.9)	82 (34.7)	141 (25.0)			82 (36.9)	81 (36.5)		
>10	98 (12.2)	21 (8.9)	77 (13.7)			21 (9.5)	20 (9.0)		
Mitotic index				0.062	0.969			0.767	0.681
≤5/50 HPF	638 (79.8)	187 (79.2)	451 (80.0)			173 (77.9)	180 (81.1)		
6‐10/50 HPF	100 (12.5)	30 (12.7)	70 (12.4)			30 (13.5)	27 (12.2)		
>10/50 HPF	62 (7.7)	19 (8.1)	43 (7.6)			19 (8.6)	15 (6.8)		
Tumor rupture				0.687	0.407			2.009	0.156
No	789 (98.6)	234 (99.2)	555 (98.4)			220 (99.1)	222 (100.0)		
Yes	11 (1.4)	2 (0.8)	9 (1.6)			2 (0.9)	0 (0.0)		
Recurrence risk^c^				11.093	0.011			0.354	0.950
Very low risk	166 (20.8)	32 (13.6)	134 (23.8)			27 (12.2)	38 (12.6)		
Low risk	260 (32.5)	87 (36.9)	173 (30.7)			78 (35.1)	81 (36.5)		
Intermediate risk	105 (13.1)	31 (13.1)	74 (13.1)			31 (14.0)	33 (14.9)		
High risk	269 (33.6)	86 (36.4)	183 (32.4)			86 (38.7)	80 (36.0)		
Adjuvant imatinib				4.367	0.037			0.502	0.479
No	583 (72.9)	160 (67.8)	423 (75.0)			146 (65.8)	153 (68.9)		
Yes	217 (27.1)	76 (32.2)	141 (25.0)			76 (34.2)	69 (31.1)		

Abbreviation: HPF, high‐powered fields.

a GB group: gastrointestinal bleeding group.

b NGB group: nongastrointestinal bleeding group.

c A risk category was assigned to all patients based on the application of the modified NIH criteria (2008 Edition).

### 3.2 Factors associated with GI bleeding

Univariate analysis identified a number of factors associated with increased odds of GI bleeding, including male gender [odds ratio(OR) = 1.572, *P* = 0.004], tumors located in the small intestine (compared to tumors located in the stomach, OR = 2.685, *P* < 0.001), and tumor size 5‐10 cm (compared to tumor size >10 cm, OR 2.132, *P* = 0.007). Patients >60 years had decreased odds of GI bleeding (OR = 0.637, *P* = 0.008). Multivariate analysis showed a similar result. Male patients (OR = 1.517, *P* = 0.011), those with tumors in the small intestine (compared to those with tumors located in the stomach, OR = 2.539, *P* < 0.001), and those with tumors 5‐10 cm (compared to tumors >10 cm, OR 2.298, *P* = 0.004) had increased odds of GI bleeding, whereas patients >60 years had decreased odds of GI bleeding (OR = 0.683, *P* = 0.031). The logistic analysis of factors associated with GI bleeding are reported in Table 2.

**Table 2:** Logistic analysis of factors associated with GI bleeding

Characteristics	Univariate			Multivariate		
	OR	95% CI	*P*	OR	95% CI	*P*
Gender						
Female	ref	–	–	ref	–	–
Male	1.572	1.152‐2.144	0.004	1.517	1.099‐2.093	0.011
Age (year)						
≤60	ref	–	–	ref	–	–
>60	0.637	0.456‐0.889	0.008	0.683	0.483‐0.966	0.031
Tumor location			<0.001			<0.001
Stomach	ref	–	–	ref	–	–
Small intestine	2.685	1.951‐3.696	<0.001	2.539	1.827‐3.526	<0.001
Colorectum	0.827	0.372‐1.840	0.641	0.746	0.332‐1.677	0.479
Tumor size (cm)			0.01			0.014
≤5	ref	–	–	ref	–	–
5‐10	1.513	1.079‐2.121	0.016	1.351	0.951‐1.919	0.094
>10	0.71	0.421‐1.196	0.198	0.588	0.342‐1.010	0.055
Mitotic index			0.969			
>5	ref	–	–			
5‐10	1.034	0.652‐1.638	0.888			
>10	1.066	0.605‐1.877	0.826			

Abbreviations: CI, confidence interval.; GI, gastrointestinal; OR, odds ratio.

### 3.3 Survival analysis

**Figure 2:**
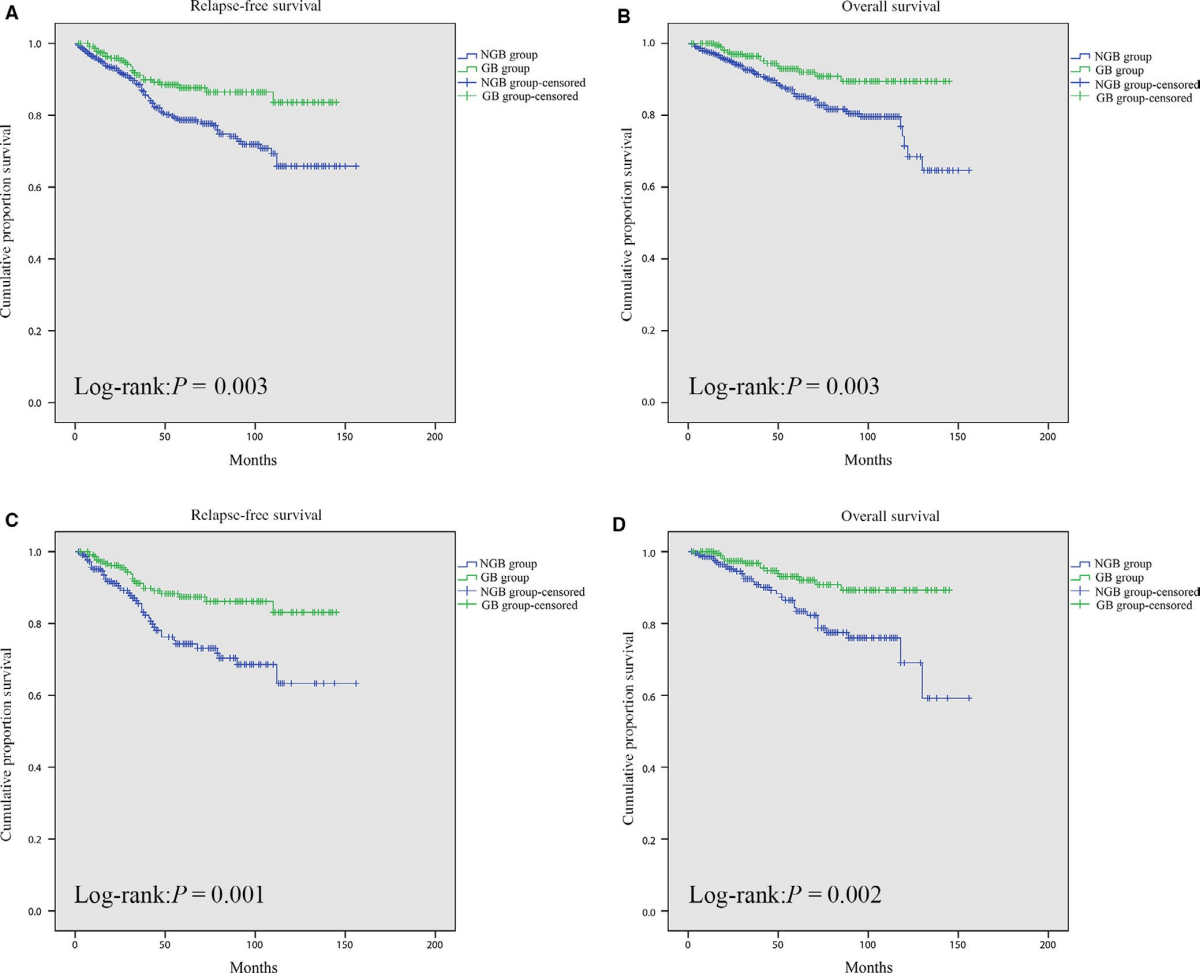
Comparison of relapse‐free survival between the GB group and NGB group before (A) and after (C) propensity score matching. Comparison of overall survival between the GB group and NGB group before (B) and after (D) propensity score matching. GB group, gastrointestinal bleeding group; NGB group, non‐gastrointestinal bleeding group

The median follow‐up time in the entire cohort was 43 (range, 3‐150) months. Before PSM, the GB group showed a better RFS (*P* = 0.003) and OS (*P* = 0.003) than the NGB group. After PSM, the 1‐, 3‐, and 5‐year RFS rates in the GB group were 97.6%, 91.2%, and 87.4%, respectively, and the corresponding rates in the NGB group were 95.1%, 85.5%, and 74.3%, respectively. The 1‐, 3‐, and 5‐year OS rates in the GB group were 99.0%, 96.8%, and 93.0%, respectively, and the corresponding rates in the NGB group were 98.6%, 91.7%, and 83.4%, respectively. RFS (*P* = 0.001) and OS (*P* = 0.002) were both superior in the GB group. Kaplan‐Meier curves are shown in Figure 2.

On multivariate Cox regression analysis, GI bleeding was identified as an independent factor associated with better RFS [hazard ratio (HR) = 0.472, *P* = 0.001] and OS (HR = 0.441, *P* = 0.005; Table 3). Age (*P* = 0.001), tumor location (*P* < 0.001), tumor size (*P* < 0.001), and mitotic index (*P* < 0.001) were statistically significant independent predictors of RFS in the multivariate analysis, and age (*P* = 0.007), tumor size (*P* = 0.004), mitotic index (*P* < 0.001), and adjuvant imatinib therapy (*P* = 0.009) were independent risk factors of OS.

**Table 3:** Univariate and multivariate relapse‐free and overall survival analysis

Characteristics	Relapse‐free survival						Overall survival					
	Univariate			Multivariate			Univariate			Multivariate		
	HR	95% CI	*P*	HR	95% CI	*P*	HR	95% CI	*P*	HR	95% CI	*P*
Gender												
Female	ref	–	–				ref	–	–			
Male	1.082	0.748‐1.564	0.677				1.135	0.740‐1.741	0.561			
Age (year)												
≤60	ref	–	–	ref	–	–	ref	–	–	ref	–	–
>60	1.75	1.210‐2.532	0.003	1.916	1.319‐2.789	0.001	1.956	1.277‐2.997	0.002	1.843	1.186‐2.865	0.007
GI‐bleeding												
No	ref	–	–	ref	–	–	ref	–	–	ref	–	–
Yes	0.578	0.371‐0.900	0.015	0.472	0.299‐0.745	0.001	0.444	0.254‐0.776	0.004	0.441	0.250‐0.776	0.005
Tumor location			<0.001			<0.001			0.041			0.056
Stomach	ref	–	–	ref	–	–	ref	–	–	ref	–	–
Small intestine	2.062	1.415‐3.007	<0.001	2.075	1.397‐3.083	<0.001	1.565	0.998‐2.454	0.051	1.561	0.974‐2.501	0.064
Colorectum	2.389	1.243‐4.580	0.009	2.600	1.339‐5.047	0.005	2.196	1.062‐4.540	0.034	2.144	1.026‐4.481	0.042
Tumor size (cm)			<0.001			<0.001			<0.001			0.004
≤5	ref	–	–	ref	–	–	ref	–	–	ref	–	–
5‐10	2.914	1.928‐4.405	<0.001	2.331	1.497‐3.630	<0.001	2.366	1.449‐3.863	0.001	2.356	1.402‐3.960	0.001
>10	3.777	2.348‐6.078	<0.001	2.128	1.267‐3.574	0.004	3.486	2.003‐6.068	<0.001	2.064	1.114‐3.824	0.021
Mitotic index			<0.001			<0.001			<0.001			<0.001
≤5/50 HPF	ref	–	–	ref	–	–	ref	–	–	ref	–	–
6‐10/50 HPF	2.749	1.735‐4.354	<0.001	2.080	1.284‐3.368	0.003	2.374	1.340‐4.208	0.003	2.153	1.187‐3.904	0.012
>10/50 HPF	4.896	2.821‐8.499	<0.001	4.243	2.357‐7.640	<0.001	6.81	3.682‐12.593	<0.001	6.966	3.558‐13.641	<0.001
Tumor rupture												
No	ref	–	–	ref	–	–	ref	–	–			
Yes	5.277	1.929‐14.435	0.001	2.697	0.945‐7.694	0.064	1.651	0.229‐11.897	0.619			
Adjuvant imatinib												
No	ref	–	–	ref	–	–	ref	–	–	ref	–	–
Yes	1.575	1.069‐2.321	0.021	0.946	0.618‐1.448	0.797	0.779	0.451‐1.345	0.37	0.465	0.262‐0.826	0.009

Abbreviations: CI, confidence interval; GI, gastrointestinal; HPF, high‐powered fields; HR, hazard ratio.

### 3.4 Subgroup analysis based on tumor location

**Figure 3:**
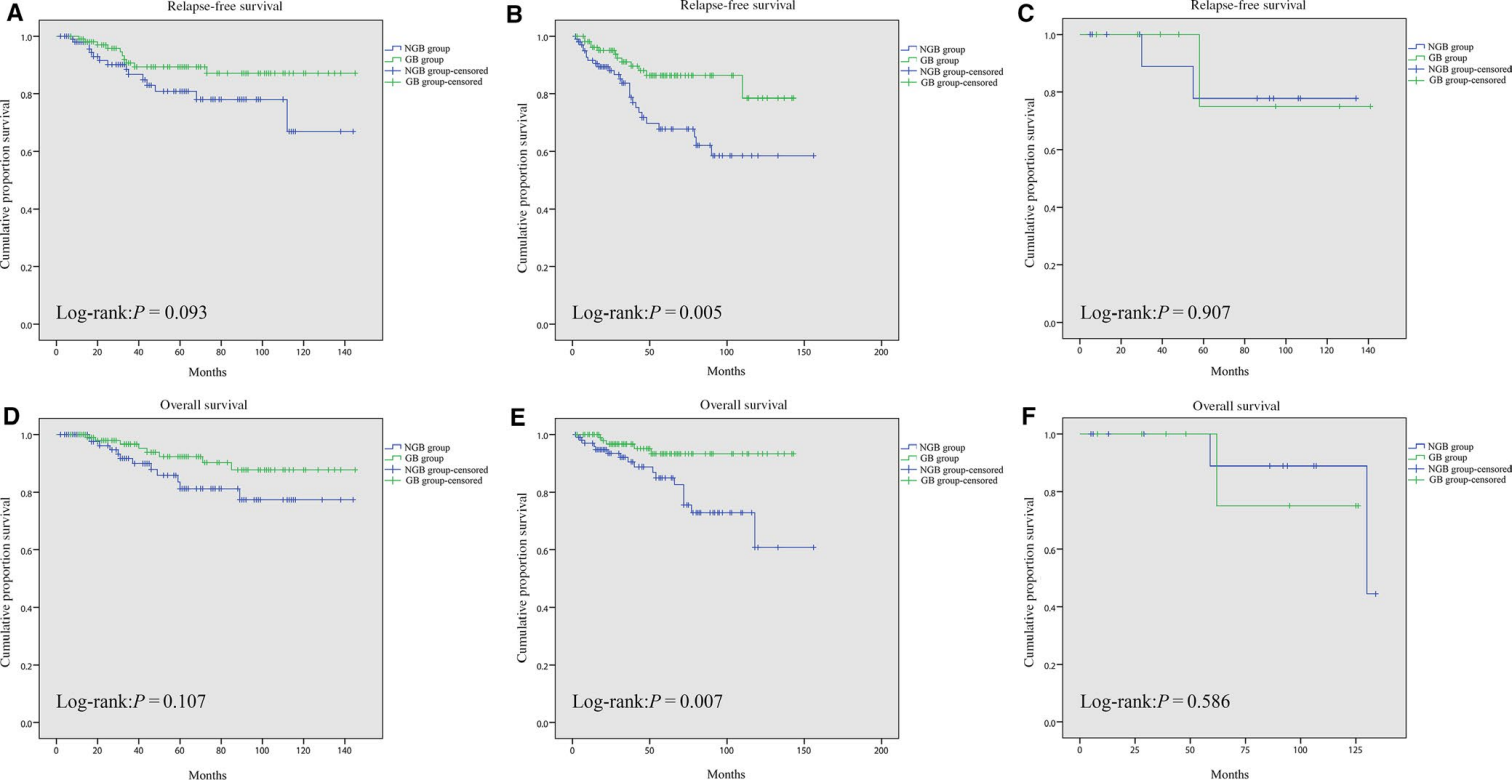
Comparison of relapse‐free survival between the GB group and NGB group with GIST located in the stomach (A), small intestine (B), and colorectum (C) after propensity score matching. Comparison of overall survival between the GB group and NGB group with GIST located in the stomach (D), small intestine (E), and colorectum (F) after propensity score matching. GB group, gastrointestinal bleeding group; NGB group, nongastrointestinal bleeding group

In the PSM groups, there were 212/444 (47.8%) patients with tumors in the stomach, 211/444 (47.5%) patients with tumors in the small intestine, and 21/444 (4.7%) patients with tumors in the colorectum. The baseline characteristics between the GB and NGB groups remained well balanced among patients with GIST derived from different regions of the GI tract (Table 4). Subgroup analysis demonstrated no significant difference in RFS and OS between the 2 groups in patients with tumors in either the stomach or the colorectum (all *P* > 0.05). However, in patients with GIST located in the small intestine, the GB group had a superior RFS (*P* = 0.005) and OS (*P* = 0.007) to the NGB group. Kaplan‐Meier curves are shown in Figure 3.

**Table 4:** Comparison of demographic and clinicopathological characteristics before and after propensity score matching in subgroup analysis

Characteristics	Stomach				Small intestine				Colorectum			
	GB^a^ group【N = 105 (%)】	NGB^b^ group【N = 107 (%)】	*χ* ^2^/*t*	*P*	GB group【N = 109 (%)】	NGB group【N = 102 (%)】	*χ* ^2^/*t*	*P*	GB group【N = 8 (%)】	NGB group【N = 13 (%)】	*χ* ^2^/*t*	*P*
Gender			0.207	0.649			0.0180	0.672			–	0.146^c^
Female	39 (37.1)	43 (40.2)			46 (42.2)	46 (45.1)			4 (50.0)	2 (15.4)		
Male	66 (62.9)	64 (59.8)			63 (61.5)	56 (54.9)			4 (50.0)	11 (84.6)		
Age (year)			0.676	0.411			0.188	0.665			–	1.000^c^
≤60	66 (62.9)	73 (68.2)			84 (77.1)	76 (74.5)			8 (100.0)	12 (92.3)		
>60	39 (37.1)	34 (31.8)			25 (22.9)	26 (25.5)			0 (0.0)	1 (7.7)		
Tumor size(cm)			0.093	0.955			0.050	0.975			3.231	0.199
≤5	56 (53.3)	57 (53.3)			59 (54.1)	54 (52.9)			4 (50.0)	10 (76.9)		
5‐10	39 (37.2)	41 (38.3)			39 (35.8)	38 (37.3)			4 (50.0)	2 (15.4)		
>10	10 (9.5)	9 (8.4)			11 (10.1)	10 (9.8)			0 (0.0)	1 (7.7)		
Mitotic index			0.464	0.793			2.920	0.232			1.477	0.478
≤5/50 HPF	81 (77.1)	85 (79.4)			85 (78.0)	86 (84.3)			7 (87.5)	9 (69.2)		
6∼10/50 HPF	15 (14.3)	12 (11.2)			15 13.8)	13 (12.7)			0 (0.0)	2 (15.4)		
>10/50 HPF	9 (8.6)	10 (9.3)			9 (8.2)	3 (2.9)			1 (12.5)	2 (15.4)		
Recurrence risk^d^			1.016	0.797			1.094	0.579			0.955	0.620
Very low risk	13 (12.4)	20 (18.7)			11 (10.1)	7 (6.9)			1 (12.5)	1 (7.6)		
Low risk	38 (36.2)	32 (29.9)			40 (36.7)	43 (42.2)			2 (25.0)	6 (46.2)		
Intermediate risk	31 (29.5)	33 (30.8)			0 (0.0)	0 (0.0)			0 (0.0)	0 (0.0)		
High risk	23 (21.9)	22 (20.6)			58 (53.2)	52 (51.0)			5 (62.5)	6 (46.2)		
Tumor rupture			–	0.495^c^			–	1.000^c^			–	–
No	104 (99.0)	107 (100.0)			108 (99.1)	102 (100.0)			8 (100.0)	13 (100.0)		
Yes	1 (1.0)	0 (0.0)			1 (0.9)	0 (0.0)			0 (0.0)	0 (0.0)		
Adjuvant imatinib			0.152	0.697			0.050	0.824			–	0.346^c^
No	72 (68.6)	76 (71.0)			70 (64.2)	67 (65.7)			4 (50.0)	10 (76.9)		
Yes	33 (31.4)	31 (29.0)			39 (35.8)	35 (34.3)			4 (50.0)	3 (23.1)		

a GB group: gastrointestinal bleeding group;

b NGB group: nongastrointestinal bleeding group;

c Fisher's exact test;

d A risk category was assigned to all patients based on the application of the modified National Institutes of Health criteria (2008 Edition).

## 4 DISCUSSION

Patients with GIST may present with a variety of nonspecific symptoms, including abdominal pain, bloating, GI bleeding, fatigue from anemia, and obstruction, depending on the site, size, and growth pattern of the tumor.^11^ About 23%‐40% of patients initially manifest with GI bleeding,^12^, ^13^, ^14^ which may be caused by ulceration or mucosal invasion.^6^, ^15^, ^16^ While “tumor rupture,” an established concept in GIST, 5, 17, 18 has been inconsistently defined as R1 resection, gross tumor spillage, and even mucosal perforation,^5^, ^19^, ^20^, ^21^, ^22^ whether GI bleeding is a kind of tumor rupture and increases the risk of recurrence or metastasis is unknown. There is insufficient evidence because studies focused on GIST patients with GI bleeding are rare, and the previously published studies are retrospective studies based on a limited sample size. This study is the first study using PSM to investigate GI bleeding in GIST, and had a relatively larger sample size. We demonstrated that GIST patients with GI bleeding have superior oncological outcomes to those without GI bleeding.

Among the entire cohort, the GB group and the NGB group showed significant differences in gender, age, tumor location, tumor size, and recurrence risk. Therefore, we conducted a logistic analysis to screen out the factors associated with GI bleeding. The analysis showed that tumors located in the small intestine were more prone to present with GI bleeding, which is consistent with the results reported by Liu et al^6^ In addition, our study found that patients with tumors 5‐10 cm in size had a higher risk of GI bleeding than patients with tumors >10 cm. This may be associated with the growth pattern of GIST, which can be defined as endoluminal, exophytic, or mixed (dumbbell‐shaped).^23^ Kang et al^24^ reported that smaller masses and lesions often protrude into the lumen, whereas larger masses and tumors often demonstrate an exophytic pattern of growth, toward the peritoneal cavity. Therefore, larger tumors may feature exophytic patterns of growth and have a lower risk of causing ulceration or mucosal invasion of the GI tract. In contrast, tumors 5‐10 cm in size might demonstrate endoluminal or mixed patterns, and therefore have a higher risk of GI bleeding.

Several retrospective studies have shown that GI bleeding was a risk factor for poor prognosis in GIST patients.^6^, ^7^, ^25^, ^26^ However, our previous study demonstrated that GI bleeding was a positive factor for RFS, and this study, which balanced the demographic data and clinicopathological characteristics between the 2 groups by PSM, showed that the GB group had superior RFS and OS. Multivariate Cox regression analysis identified age, tumor location, tumor size, mitotic index, and adjuvant imatinib treatment as independent risk factors of prognosis, which was consistent with previous studies.^5^, ^27^, ^28^, ^29^ Moreover, it also found that GI bleeding was a positive prognostic factor. Therefore, GI bleeding in GIST patients does not appear to act like tumor rupture or tumor necrosis, which is associated with poor clinical outcomes.^5^, ^30^ Additionally, as GISTs derived from different parts of the GI tract have different malignancy potentials^31^, ^32^, ^33^ and varying risks of GI bleeding, we conducted subgroup analysis based on tumor location. The log‐rank test revealed that the GB group had a superior outcome to the NGB group in GIST of the small intestine, whereas the difference in prognosis between the 2 groups was insignificant in GISTs of the stomach and colorectum.

The better outcomes of the GB group used to be attributed to the smaller size of the tumor.^8^ However, this study eliminated the difference in tumor size between the 2 groups and found that the superior outcome of the GB group was mainly because of improved outcomes in small intestine GIST. Considering that GI bleeding might make patients more vigilant than other nonspecific symptoms, such as abdominal pain or bloating, and the smaller cavity channel of the small intestine might cause an earlier presentation of bleeding than other locations, this special symptom could induce earlier medical treatment, thereby generating a superior outcome in small intestine GIST. Whether GIST with higher bleeding risk has a lower aggressive ability and whether GI bleeding can be a potential factor for risk classification of GIST needs further study.

It is infeasible to conduct a prospective randomized study to compare the clinicopathological characteristics and prognosis between GIST patients with and without GI bleeding. Therefore, PSM, which is widely used in retrospective studies,^32^, ^33^, ^34^ was performed here to eliminate the confounders between the 2 groups. Though some potential factors that influence the outcome of GIST patients may exist that were not included in the calculation of the propensity score, to the best of our knowledge, this study is the most sophisticated study focusing on GI bleeding in GIST patients. However, this was a study at a single center with a relatively limited number of patients, and a larger multicenter study is needed to verify its conclusions.

## 5 CONCLUSIONS

In summary, GIST patients with age < 60, male gender, tumors located in the small intestine, and tumors 5‐10 cm in size were more likely to manifest with GI bleeding. Compared with the NGB group, the GB group had a superior RFS and OS. This difference was statistically significant in small intestine GIST, but not in stomach or colorectal GIST.

## CONFLICT OF INTEREST

The authors declare that there is no conflict of interest.

## Data Availability

All data included in this study are available upon request by contact with the corresponding author.

## References

[1] Joensuu H , Hohenberger P , Corless CL . Gastrointestinal stromal tumour. Lancet. 2013;382:973‐983. 23623056 10.1016/S0140-6736(13)60106-3

[2] Mucciarini C , Rossi G , Bertolini F , et al. Incidence and clinicopathologic features of gastrointestinal stromal tumors: a population‐based study. BMC Cancer. 2007;20:230. 10.1186/1471-2407-7-230 PMC2241631 18096058

[3] Nilsson B , Bümming P , Meis‐Kindblom JM , et al. Gastrointestinal stromal tumors: The incidence, prevalence, clinical course, and prognostication in the preimatinib mesylate era—a population‐based study in western Sweden. Cancer. 2005;103:821‐829. 15648083 10.1002/cncr.20862

[4] Joensuu H . Risk stratification of patients diagnosed with gastrointestinal stromal tumor. Hum Pathol. 2008;39:1411‐1419. 18774375 10.1016/j.humpath.2008.06.025

[5] Rutkowski P , Nowecki ZI , Michej W , et al. Risk criteria and prognostic factors for predicting recurrences after resection of primary gastrointestinal stromal tumor. Ann Surg Oncol. 2007;14:2018‐2027. 17473953 10.1245/s10434-007-9377-9

[6] Liu QI , Li Y , Dong M , Kong F , Dong QI . Gastrointestinal bleeding is an independent risk factor for Poor Prognosis in GIST patients. Biomed Res Int. 2017;2017:7152406. 28589146 10.1155/2017/7152406 PMC5447278

[7] Huang Y , Zhao R , Cui Y , et al. Effect of gastrointestinal bleeding on gastrointestinal stromal tumor patients: a retrospective cohort study. Med Sci Monit. 2018;24:363‐369. 29346334 10.12659/MSM.908186 PMC5784333

[8] Yin Z , Gao J , Liu W , et al. Clinicopathological and prognostic analysis of primary gastrointestinal stromal tumor presenting with gastrointestinal bleeding: a 10‐year retrospective study. J Gastrointest Surg. 2017;21:792‐800. 28275959 10.1007/s11605-017-3385-2

[9] Rosenbaum PR , Rubin DB . The central role of the propensity score in observational studies for causal effects. Biometrika. 1983;70:41‐55.

[10] Austin PC . Balance diagnostics for comparing the distribution of baseline covariates between treatment groups in propensity‐score matched samples. Stat Med. 2009;28:3083‐3107. 19757444 10.1002/sim.3697 PMC3472075

[11] Rubin BP , Heinrich MC , Corless CL . Gastrointestinal stromal tumour. Lancet. 2007;369:1731‐1741. 17512858 10.1016/S0140-6736(07)60780-6

[12] Scarpa M , Bertin M , Ruffolo C , Polese L , D'Amico DF , Angriman I . A systematic review on the clinical diagnosis of gastrointestinal stromal tumors. J Surg Oncol. 2008;98:384‐392. 18668671 10.1002/jso.21120

[13] Wang M , Xu J , Zhang Y , et al. Gastrointestinal stromal tumor: 15‐ years’ experience in a single center. BMC Surg. 2014;14:93. 25403624 10.1186/1471-2482-14-93 PMC4254179

[14] Rammohan A , Sathyanesan J , Rajendran K , et al. A gist of gastrointestinal stromal tumors: a review. World J Gastrointest Oncol. 2013;5:102‐112. 23847717 10.4251/wjgo.v5.i6.102 PMC3708046

[15] Miettinen M , Sobin LH , Lasota J . Gastrointestinal stromal tumors of the stomach: a clinicopathologic, immunohistochemical, and molecular genetic study of 1765 cases with long‐term follow‐up. Am J Surg Pathol. 2005;29:52‐68. 15613856 10.1097/01.pas.0000146010.92933.de

[16] Trupiano JK , Stewart RE , Misick C , Appelman HD , Goldblum JR . Gastric stromal tumors: a clinicopathologic study of 77 cases with correlation of features with nonaggressive and aggressive clinical behaviors. Am J Surg Pathol. 2002;26:705‐714. 12023574 10.1097/00000478-200206000-00003

[17] Kitabayashi K , Seki T , Kishimoto K , et al. A spontaneously ruptured gastric stromal tumor presenting as generalized peritonitis: report of a case. Surg Today. 2001;31:350‐354. 11321348 10.1007/s005950170159

[18] Cegarra‐Navarro MF , de la Calle MA , Girela‐Baena E , et al. Ruptured gastrointestinal stromal tumors: radiologic findings in six cases. Abdom Imaging. 2005;30:535‐542. 15834676 10.1007/s00261-005-0308-6

[19] Joensuu H , Vehtari A , Riihimäki J , et al. Risk of recurrence of gastrointestinal stromal tumour after surgery: an analysis of pooled population‐based cohorts. Lancet Oncol. 2012;13:265‐274. 22153892 10.1016/S1470-2045(11)70299-6

[20] Takahashi T , Nakajima K , Nishitani A , et al. An enhanced risk‐group stratifcation system for more practical prognostication of clinically malignant gastrointestinal stromal tumors. Int J Clin Oncol. 2007;12:369‐374. 17929119 10.1007/s10147-007-0705-7

[21] Tryggvason G , Gíslason HG , Magnússon MK , Jónasson JG . Gastrointestinal stromal tumors in Iceland, 1990–2003: the Icelandic GIST study, a population based incidence and pathologic risk stratification study. Int J Cancer. 2005;117:289‐293. 15900576 10.1002/ijc.21167

[22] Hølmebakk T , Bjerkehagen B , Boye K , Bruland Ø , Stoldt S , Sundby Hall K . Definition and clinical significance of tumour rupture in gastrointestinal stromal tumours of the small intestine. Br J Surg. 2016;103:684‐691. 26988241 10.1002/bjs.10104

[23] Min YW , Park HN , Min B‐H , Choi D , Kim K‐M , Kim S . Preoperative predictive factors for gastrointestinal stromal tumors: analysis of 375 surgically resected gastric subepithelial tumors. J Gastrointest Surg. 2015;19:631‐638. 25472028 10.1007/s11605-014-2708-9

[24] Kang HC , Menias CO , Gaballah AH , et al. Beyond the GIST: mesenchymal tumors of the stomach. Radiographics. 2013;33:1673‐1690. 24108557 10.1148/rg.336135507 PMC3794320

[25] Lv A , Li Z , Tian X , et al. SKP2 high expression, KIT exon 11 deletions, and gastrointestinal bleeding as predictors of poor prognosis in primary gastrointestinal stromal tumors. PLoS ONE. 2013;8:e62951. 23690967 10.1371/journal.pone.0062951 PMC3656858

[26] Wang H , Chen P , Liu X‐X , et al. Prognostic impact of gastrointestinal bleeding and expression of PTEN and Ki‐67 on primary gastrointestinal stromal tumors. World J Surg Oncol. 2014;12:89. 24712384 10.1186/1477-7819-12-89 PMC3991912

[27] DeMatteo RP , Gold JS , Saran L , et al. Tumor mitotic rate, size, and location independently predict recurrence after resection of primary gastrointestinal stromal tumor (GIST). Cancer. 2008;112:608‐615. 18076015 10.1002/cncr.23199

[28] Yang Z , Feng X , Zhang P , et al. Clinicopathological outcomes and pprognosis of elderly patients (≥ 65 years) with gastric gastrointestinal stromal yumors (GISTs) undergoing curative‐intent resection: a multicenter data review. J Gastrointest Surg. 2018;23:904–913. 10.1007/s11605-018-3944-1. 30324400

[29] Joensuu H , Eriksson M , Sundby Hall K , et al. One vs three years of adjuvant imatinib for operable gastrointestinal stromal tumor: a randomized trial. JAMA. 2012;307:1265‐1272. 22453568 10.1001/jama.2012.347

[30] Liu X , Qiu H , Zhang P , et al. Prognostic role of tumor necrosis in patients undergoing curative resection for gastric gastrointestinal stromal tumor: a multicenter analysis of 740 cases in China. Cancer Med. 2017;6:2796‐2803. 29058376 10.1002/cam4.1229 PMC5727342

[31] Gold JS , Gönen M , Gutiérrez A , et al. Development and validation of a prognostic nomogram for recurrence‐free survival after complete surgical resection of localised primary gastrointestinal stromal tumour: a retrospective analysis. Lancet Oncol. 2009;10:1045‐1052. 19793678 10.1016/S1470-2045(09)70242-6 PMC3175638

[32] Nakagawa N , Kanda M , Ito S , et al. Pathological tumor infiltrative pattern and sites of initial recurrence in stage II/III gastric cancer: Propensity score matching analysis of a multi‐institutional dataset. Cancer Med. 2018;7:6020‐6029. 30411544 10.1002/cam4.1868 PMC6308072

[33] Shen J , Wen J , Li C , et al. The prognostic value of microvascular invasion in early‐intermediate stage hepatocelluar carcinoma: a propensity score matching analysis. BMC Cancer. 2018;18:278. 29530006 10.1186/s12885-018-4196-x PMC5848587

[34] Piedimonte S , Richer L , Souhami L , et al. Clinical significance of isolated tumor cells and micrometastasis in low‐grade, stage I endometrial cancer. J Surg Oncol. 2018;118:1194‐1198. 30353577 10.1002/jso.25259

